# Pathogen Metagenomics Reveals Distinct Lung Microbiota Signatures Between Bacteriologically Confirmed and Negative Tuberculosis Patients

**DOI:** 10.3389/fcimb.2021.708827

**Published:** 2021-09-13

**Authors:** Li Ding, Yanmin Liu, Xiaorong Wu, Minhao Wu, Xiaoqing Luo, Hui Ouyang, Jinyu Xia, Xi Liu, Tao Ding

**Affiliations:** ^1^Program of Infectious Diseases, The Fifth Affiliated Hospital of Sun Yat-sen University, Zhongshan School of Medicine, Sun Yat-sen University, Guangzhou, China; ^2^Key Laboratory of Tropical Disease Control (Sun Yat-sen University), Ministry of Education, Guangzhou, China; ^3^Department of Infectious Diseases, The Fifth Affiliated Hospital of Sun Yat-sen University, Zhuhai, China; ^4^Department of Immunology, Zhongshan School of Medicine, Sun Yat-sen University, Guangzhou, China

**Keywords:** tuberculosis, pathogen metagenomics sequence, lung microbiota, bronchoalveolar lavage fluid, bacteriological confirmation, distinct tuberculosis lung microbiota signatures

## Abstract

Understanding the dynamics of lung microbiota in tuberculosis patients, especially those who cannot be confirmed bacteriologically in clinical practice, is imperative for accurate diagnosis and effective treatment. This study aims to characterize the distinct lung microbial features between bacteriologically confirmed and negative tuberculosis patients to understand the influence of microbiota on tuberculosis patients. We collected specimens of bronchoalveolar lavage fluid from 123 tuberculosis patients. Samples were subjected to metagenomic next-generation sequencing to reveal the lung microbial signatures. By combining conventional bacterial detection and metagenomic sequencing, 101/123 (82%) tuberculosis patients were bacteriologically confirmed. In addition to *Mycobacterium tuberculosis*, *Staphylococcus aureus*, *Kluyveromyces lactis*, and *Pyricularia pennisetigena* were also enriched in the bacteriological confirmation group. In contrast, *Haemophilus parainfluenzae* was enriched in the bacteriologically negative group. Besides, microbial interaction exhibits a different state between bacteriologically confirmed and negative tuberculosis patients. *Mycobacterium tuberculosis* was confirmed correlated with clinical characteristics such as albumin and chest cavities. Our study comprehensively demonstrates the correlation between unique features of lung microbial dynamics and the clinical characteristics of tuberculosis patients, suggesting the importance of studying the pulmonary microbiome in tuberculosis disease and providing new insights for future precision diagnosis and treatment.

## Introduction

Tuberculosis (TB), caused by *Mycobacterium tuberculosis* (Mtb), is a severe infectious and leading deathly disease worldwide (WHO, 2019). Clinical diagnosis and treatment of TB is complicated and controversial. Bacteriological confirmation (BC) in TB refers to the identification of Mtb in the sputum smear microscopy or sputum culture from patients and is widely used clinically for TB diagnosis. The World Health Organization (WHO) reported that 55% of TB patients were BC in 2018. Those bacteriologically negative (BN) patients were mainly diagnosed based on clinical symptoms or abnormalities on chest radiography or suggestive histology (Pek et al., 2002; Muttamba et al., 2019). Zhang et al. found that the cavity on the chest radiography and diabetes comorbidity were significantly overrepresented in the BC group (Zhang et al., 2020). However, it remains largely unknown whether lung microbes differ between BC and BN patients.

Lung and the entire lower respiratory tract (LRT) inhabit niches-specific microbial communities (Man et al., 2017; Naidoo et al., 2019). For example, *Tropheryma whipplei*, which causes Whipple disease and primarily exists in gut, is found in the LRT but not the upper respiratory tract (URT), suggesting that the LRT provides a distinct microbial habitats (Qin, 2016). In fact, the microbial biomass of LRT is significantly less than that of the URT due to nutrient scarcity and local immune clearance (Dickson and Huffnagle, 2015). To date, limited studies have investigated the lung microbial feature of TB patients. Zhou et al. found that the genus *Cupriavidus* was significantly enriched in TB patients compared to healthy individuals ( Zhou et al., 2015). Hu et al. found the family *Bacillaceae* and the genus *Anoxybacillus* were enriched in the lungs of the BC patients regardless of the effect of antibiotics (Hu et al., 2020). These two studies, which were both based on 16S rRNA sequencing, showed that bacterial composition varied between TB patients and healthy individuals. Due to the high prevalence of bacteria and the fact that the tuberculosis pathogen itself is a bacterium, these recent lung microbiome studies focused on the bacteria (Man et al., 2017). In fact, fungal microbiota, or mycobiome, is also an important part of lung microbiome and interacts with bacteria impacting host immunity. Since the condition may go unnoticed, fungal infections are very dangerous for TB patients. Amiri et al. found 16 of 130 (12.3%) TB patients were tested fungi positive, with high prevalence of *Aspergillus fumigatus* (10/16, 62.5%) and *Candida albicans* (6/16, 37.5%)in fungi culture, indicating fungi should be considered as a major coinfection agent in TB (Amiri et al., 2018).

Metagenomic next-generation sequencing (mNGS) is highly sensitive to detect various microorganisms, and thus has been now applied to the diagnosis of infectious diseases, especially with unidentified pathogens (Wilson et al., 2014; Miao et al., 2018). mNGS is now becoming a part of the diagnostic workup in TB patients for its superior sensitivity, speed, and cost-effectiveness.

In the present study, we applied the mNGS approach to systematically study the characteristic differences in lung microbiota between patients in the BC and BN groups to understand the impacts of microbiota on TB patients (Matarazzo et al., 2012; Wylie, 2017; Li et al., 2019). We improved the bacteriological confirmation approach by combining the traditional culture method with mNGS and identified the unique microbial features that link to the outcomes of bacteriological confirmation. Our study provides a preliminary discussion of the roles of these microbes in the progress of TB as well as a novel perspective on understanding the various phenotypes of TB disease.

## Materials and Methods

### Study Patients

**Figure 1:**
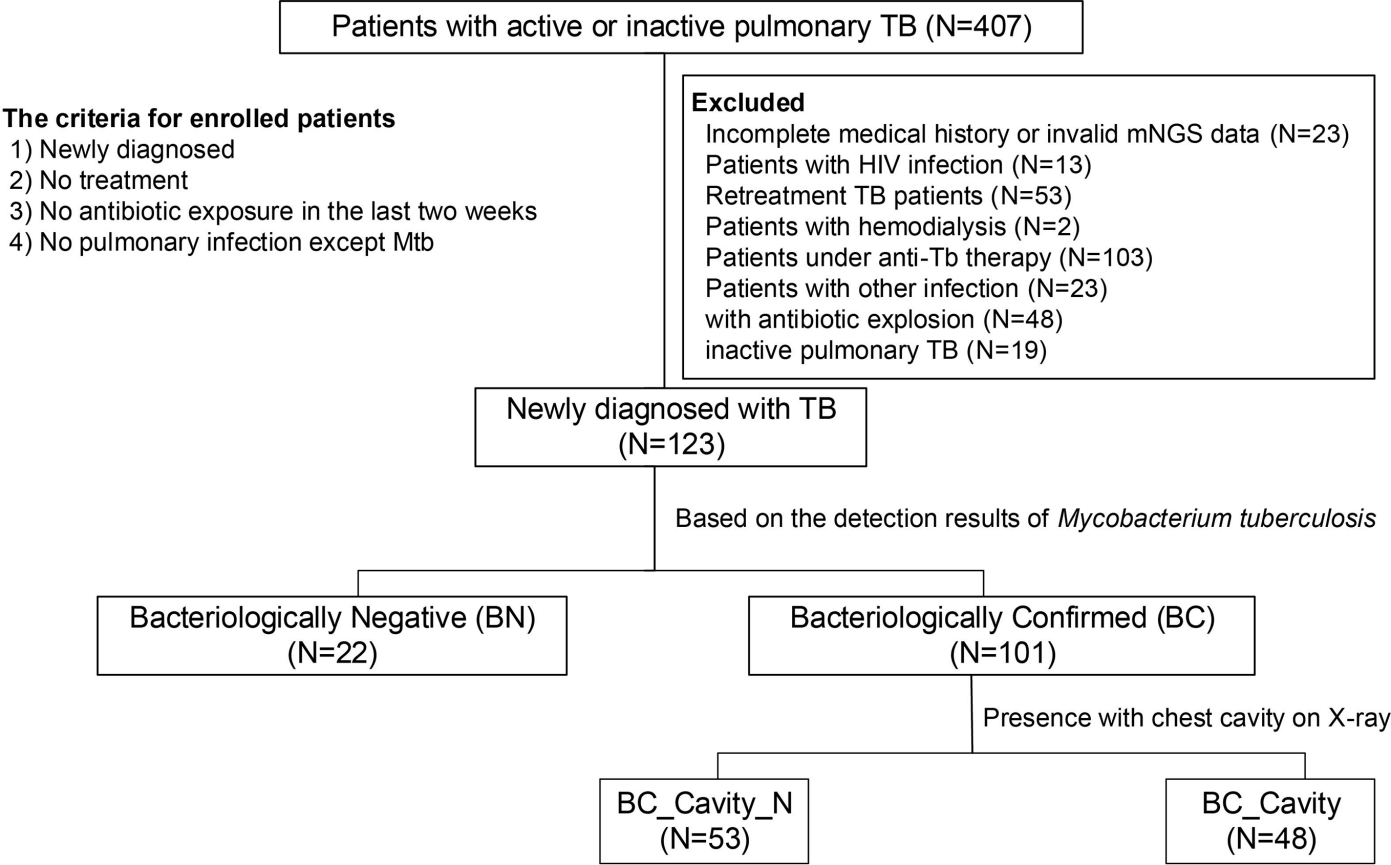
Criteria for the enrollment of TB patients in the present study. From a total of 407 diagnosed patients, 123 patients were included in this study. Enrollment criteria included 1) new diagnosis, 2) no treatment, 3) no antibiotic exposure in the last two weeks, and 4) no other pulmonary infection except tuberculosis. The enrolled patients were divided into two groups, bacteriologically confirmed (or BC, N = 101) and bacteriologically negative (or BN, N = 22), based on an improved method in which we combined traditional culture-based approach with mNGS. BC patients were further categorized as BC_Cavity (N = 48) and BC_Cavity_N (N = 53) based on the presence of cavity on the chest radiography.

A total of 407 patients were diagnosed at the Fifth Affiliated Hospital of Sun Yat-sen University between April 2018 and April 2019. Following strict criteria as described (**Figure 1**), a total of 123 patients newly diagnosed with TB were enrolled in the study. This study was approved by the Institutional Review Board of the Fifth Affiliated Hospital, Sun Yat-sen University, and written informed consent was obtained from all participants. The enrolled patients were categorized into two groups, bacteriologically confirmed (BC) and bacteriologically negative (BN). BC refers to those patients whose bronchoalveolar lavage fluid (BALF) was detected of Mtb by metagenomic sequencing or traditional detection method, including sputum smear, culture, and NAAT. BN refers to those who were diagnosed as tuberculosis clinically but Mtb cannot be detected in respiratory samples by either mNGS or traditional sputum check.

### Sample Processing and DNA Extraction

BALF was collected from the sick lung of each patient using bronchoscopy according to standard procedures. Samples of 3–5 ml BAL were immediately stored in −80°C freezers and processed within three days of collection. DNA was extracted according to the instructions of the kit (DP316, Tiangen Biotech, Beijing, China), which was completed in BGI China.

### Pathogen Metagenomics Sequencing

DNA libraries were constructed using the end-repair method, including the adapter addition and polymerase chain reaction (PCR) amplification. The library was validated by 2100 Bioanalyzer system (Agilent Technologies, Inc.) then was sequenced on the Ion Torrent next-generation sequencing (BGISEQ100, BGI China) platform. The metagenomic sequencing data had been deposited to the Dryad Archive database (https://datadryad.org/stash/share/B5qwEaAtFy_UCDhM_24JjAJLyK6vg8UYHzgF1Qqzyto).

### Sequence Taxonomic Annotation

We assigned the reads to taxonomy using k-mer-based algorithms according to the Kraken2 taxonomic annotation pipeline (Wood et al., 2019). The database consisted of bacterial, fungal, viral, and archaea reference genomes from NCBI (http://www.ncbi.nlm.nih.gov) and FungiDB (http://fungidb.org). We built the reference libraries by count distinct 31-mer to obtain high accuracy and fast classification speeds. The produced metagenomic read counts were normalized in each kingdom respectively (Coker et al., 2019).

### Statistical Analysis

We applied the linear discriminant analysis effect size (LEfSe) method to identify differential species based on relative abundance, which was implemented on the Galaxy platform (http://huttenhower.sph.harvard.edu/galaxy). Other statistical analyses were performed in R platform.

## Results

### Demographics and Clinical Characteristics of Study Patients

A total of 407 patients were diagnosed with tuberculosis at the Fifth Affiliated Hospital of Sun Yat-sen University between April 2018 and April 2019. Of these, 123 patients were enrolled in this study following strict criteria, including 1) new diagnosis, 2) no treatment, 3) no antibiotic exposure in the last two weeks, 4) no pulmonary infection except Mtb (**Figure 1**). By combining the results of conventional bacterial detection and mNGS, 101 (82%) TB patients in our cohort were identified as bacteriologically confirmed (BC) (Liu et al., 2020); the BC rate is higher that of the WHO 2019 report (55%) (WHO, 2019). Significant differences were detected in cavity on the chest radiography, monocyte, and alanine aminotransferase (ALT) between BC and BN groups (**Table 1**). The BC group had a higher rate of cavity and high-monocyte than the BN group (cavity: 47.52 *vs* 4.55%; monocyte: 44.55 *vs* 18.18%, both *p* < 0.05). The ALT concentration of BC (median: 13; IQR: 9–21) was significantly lower than that of BN (median: 19.5; IQR:13.25–25.5). No significant difference was found in age, gender, and other metrics between the BC and BN groups.

**Table 1:** Demographics and clinical characteristics of study patients.

	BC (N = 101)	BN (N = 22)	p-value
Age, mean (SD), year	43.58 (15.32)	50.18 (16.18)	1.00
Gender, Female/Male (%)	29/72 (28.71/71.29)	8/14 (36.37/63.63)	0.65
BMI, mean (SD), kg/m^2^	21.15 (3.11)	21.78 (1.90)	0.22
Smoking, N/Y (%)	66/35 (65.35/34.65)	14/8 (63.64/36.36)	1.00
Diabetes, N/Y (%)	77/24 (76.24/23.76)	20/2 (90.91/9.09)	0.22
Cavity, N/Y (%)	53/48 (52.48/47.52)	21/1 (95.45/4.55)	0.00*
WBC, mean (SD),	7.01 (2,42)	6.75 (3.24)	0.72
CD4, mean (SD),	592 (249)	597 (208)	0.92
CD8, mean (SD),	362 (188)	349 (151)	0.75
Hb, mean (SD),	135.26 (18.57)	138.00 (16.66)	0.49
VitD, mean (SD),	27.27 (12.40)	36.17 (29.57)	0.27
creatinine, mean (SD),	69.04 (21.20)	68.73 (14.55)	0.93
TP, mean (SD),	68.69 (6.59)	70.24 (7.62)	0.39
ALB, mean (SD),	40.73 (5.69)	42.4 (7.33)	0.32
globulin, mean (SD),	27.96 (4.99)	27.84 (6.06)	0.93
LDL, mean (SD),	2.54 (0.76)	2.51 (0.85)	0.87
HDL, median (IQR),	1 (0.85–1.29)	1.15 (0.92–1.38)	0.27
LDH, mean (SD),	171.33 (45.89)	170.47 (31.83)	0.92
NEU, median (IQR),	4.29 (3.28–4.29)	3.39 (2.54–5.19)	0.20
urea, median (IQR),	4.3 (3.6–5.2)	4.5 (3.58–5.78)	0.45
ALT, median (IQR),	13 (9–21)	19.5 (13.25–25.5)	0.03*
AST, median (IQR),	20 (15–26)	21 (19.25–26)	0.11
Tbil, median (IQR),	10.6 (7.9–14.4)	10.05 (7.00–15.55)	0.83
Dbil, median (IQR),	4.3 (3.0–5.4)	3.8 (2.2–6.28)	0.71
TC, median (IQR),	4.25 (3.68–4.99)	4.07 (3.88–5.15)	0.57
Trig, median (IQR),	0.94 (0.73–1.27)	0.93 (0.73–1.27)	0.93
CRP, median (IQR),	6.45 (0.5–21.12)	13.08 (0.24–15.43)	0.77
ESR, median (IQR),	22 (9.6–47)	16 (8–52)	0.61
monocyte-high, N (%)	45 (44.55)	4 (18.18)	0.04*
GRF-normal, N (%)	60 (59.41)	12 (54.55)	0.92
GRF-moderately reduced, N (%)	33 (32.67)	8 (36.36)	
GRF-Severely reduced, N (%)	8 (7.92)	2 (9.09)	

SD, standard deviation; BMI, body mass index; IQR, interquartile range; Cavity, cavity on the chest X-ray; WBC, white blood cell; Hb, hemoglobin; VitD, vitamin D; TP, total protein; ALB, albumin; HDL, high density lipoprotein; LDL, low density lipoprotein; LDH, lactate dehydrogenase; NEU, neutrophil; Lym, lymphocyte; ALT, alanine aminotransferase; AST, aspartate transferase; Tbil, total bilirubin; Dbil, direct bilirubin; TC, total cholesterol; Trig, triglyceride; CRP, c-reaction protein; ESR, erythrocyte sedimentation rate; GRF, growth hormone releasing factor; *P < 0.05.

According to the presence of cavity on the chest radiography, the BC patients were categorized as BC_Cavity (N = 48) and BC_Cavity_N (N = 53). Significant differences in diabetes, total protein (TP), albumin (ALB), and monocyte were found between these two groups (*p* < 0.05).

### The lung Microbial Signature Significantly Differed Between BC and BN

After quality filtering, we obtained a total of 136,601,563 metagenomic sequencing reads, the majority of which are of human origin. After removing those of human source, we assigned the remaining reads to the taxonomy of microorganisms (including bacteria, fungi, virus, and archaea). To improve the quality of subsequent analysis, we removed those species with less than 10 reads assigned.

**Figure 2:**
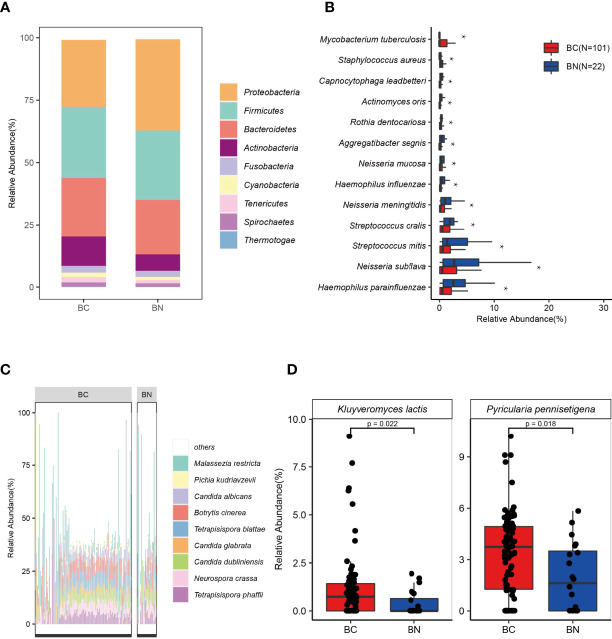
The lung microbial signature differed between BC and BN patients. Stacked bar chart indicates that the distribution of bacteria in the BC and BN **(A)**. *Proteobacteria*, *Firmicutes*, and *Bacteroidetes* were the three most abundant phyla among TB patients **(A)**. Bacteria that were significantly enriched in BC or BN were identified using LEfSe, of which those with the highest relative abundance were shown in **(B)**. *Tetrapisispora blattae*, *Candida albicans*, *Malassezia restricta*, *Tetrapisispora phaffii*, *Neurospora crass*, and *Botrytis cinerea* were the most abundant fungal species in TB patients **(C)**. Two fungal species were significantly enriched in the BC patients **(D)** * represents p value < 0.05.

The dominant parts of the classified reads were bacteria (97%). We identified 39 phyla of bacteria from all samples, with *Proteobacteria*, *Firmicutes*, and *Bacteroidetes* as the three most abundant phyla. Regarding the differences in the relative abundance present in the BC and BN groups, *Proteobacteria* (BN: 36.50% *vs* BC: 26.77%, *p* < 0.05) was more abundant in the BN group, while *Actinobacteria* (BC: 11.81% *vs* BN: 6.64%) was more abundant in the BC group (**Figure 2A**). To further explore the differences between the two groups at the species level, we applied the Linear discriminant analysis Effect Size (LEfSe) method and found a total of 116 species were significantly differentially enriched in either BC or BN (LDA score >2.0, *p* < 0.05): 99 bacterial species were enriched in BC and 17 bacterial species were enriched in BN (**Supplementary Table S1**). Of these, 13 bacteria had an average relative abundance greater than 1%. *Mycobacterium tuberculosis* (BC: 6.88% *vs* BN: 0%, *p* < 0.05), which belongs to *Actinobacteria*, was detected only in BC group. *Haemophilus parainfluenzae* (BC: 1.48% *vs* BN: 3.19%, *p* < 0.05) and *Neisseria subflava* (BC: 3.17% *vs* BN: 5.36%, *p* < 0.05), which belong to *Proteobacteria*, were the species with higher relative abundance in BN group than in BC group (**Figure 2B**).

Of all the microbial reads, 1.7% were classified as fungi. *Ascomycota* (relative abundance in fungi—BC: 85.95% *vs* BN: 78.69%), *Basidiomycota* (BC: 13.59% *vs* BN: 21.15%), and *Microsporidia* (BC: 0.46% *vs* BN: 0.16%), were the three most dominant phyla. At the species level, *Malassezia restricta* (BC: 8.91% *vs* BN: 16.27%), *Candida albicans* (BC: 5.56% *vs* BN: 4.75%), *Botrytis cinerea* (BC: 5.97% *vs* BN: 4.52%), *Tetrapisispora blattae* (BC: 4.66% *vs* BN: 2.80%, *p* < 0.05), and *Tetrapisispora phaffii* (BC: 4.41% *vs* BN: 3.45%) were the five fungal species with highest relative abundance (**Figure 2C**). *M. restricta*, an opportunistic pathogen which can be found in patients with cystic fibrosis (Willger et al., 2014), were detected in 80 patients with TB (65%). Notably, the average relative abundance of *M. restricta* in BN was higher than that in BC, although its occurrence rates were not significantly different between these two groups. LEfSe analysis identified that two fungal species, *Pyricularia pennisetigena* and *Kluyveromyces lactis*, were significantly enriched in the BC group (**Figure 2D**).

We found *Euryarchaeota*, *Crenarchaeota*, and *Thaumarchaeota* were the three major phyla of archaea. No significant difference at the phyla level was found between BC group and BN group (**Figure S1A**). *Euryarchaeota* was the main archaea in both groups (BC: 80.12% *vs* BN: 83.53%). At the species level, *Methanosarcina barkeri*, *Methanococcus maripaludis*, and *Methanococcus aeolicus* were the three dominant species in the *Euryarchaeota*, and their relative abundance was not significantly different between the two groups. Only the relative abundance of *Sulfolobus islandicus* of the phylum of *Crenarchaeota* differed significantly between the two groups (**Figure S1B**).

The presence of viruses in these lung microbiome samples is rare and easily neglected; however we still observed some interesting results. *Siphoviridae* was the most abundant family in the lung of patients with TB, and its relative abundance in BN group (29.66%) is higher than that in BC group (15.11%) (**Figure S1C**). The family of *Siphoviridae* mainly includes various phages, such as *Streptococcus*-associated phage and *Staphylococcus*-associated phage. In addition, human herpesvirus, including *Human betaherpesvirus 6B*, *Human alphaherpesvirus 3*, was another common type of virus in the lung of patients with TB. These results suggest that attention should be paid to the presence of various types of viruses in the lungs during the treatment of TB.

### Microbial Interactions Exhibited Different States Between BC and BN Patients

**Figure 3:**
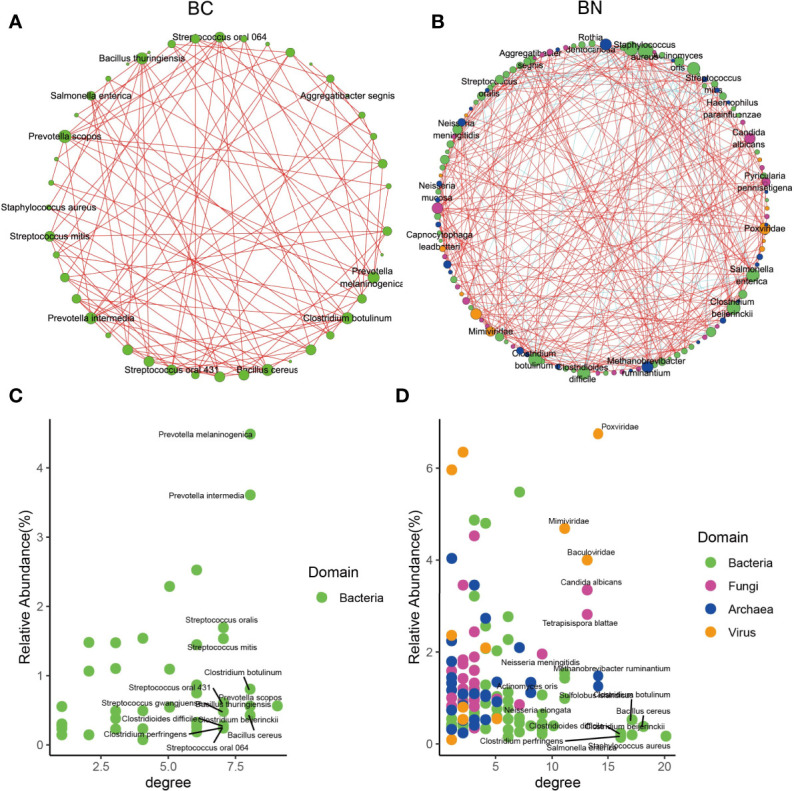
Microbial interactions exhibited different state between BC and BN patients. Microbial interaction networks were calculated as the Spearman’s correlation of the relative abundance of the microbes and visualized using Cytoscape for the BC **(A)** and BN **(B)** patients respectively. Only those microbes with relative abundance more than 0.1% and presenting at least 50% of samples were included in the plots. The species with the highest average relative abundance and degree in the BC and BN networks are shown in **(C, D)** respectively.

To determine how these pulmonary microbes interact with each other and whether these interactions are associated with the TB phenotype, we used Spearman correlations to compare the co-occurrence network of microbes with relative abundance above 0.1% and present in at least 50% of samples between the BC and BN groups. The results showed that the interaction between microorganisms was decreased in the BC group compared to that in the BN group. After excluding those with correlations less than 0.7 or p-FDR values greater than 0.05, 44 interaction nodes and 108 connections were retained in the BC network, while 122 interaction nodes and 324 connections were retained in the BN network (**Figures 3A, B**). Notably, all the remaining nodes in the BC group were bacteria, while in the BN group bacteria interacted with other kingdoms of microorganisms as well. These results suggested that the microbial interaction network was more stable and diverse in the BN group than in the BC group. Interaction nodes in the BN group showed significantly higher degrees, implying that they had more connections with other microorganisms than in the BC group (**Figures 3C, D**). Remarkably, *Neisseria* and *Actinomyces* had high degrees in the BN group, while *Prevotella*, *Streptococcus*, and *Haemophilus parainfluenzae*, common respiratory bacteria (Popov et al., 2019; Sierra et al., 2020) had high degrees in the BC group. These species with high degrees are commonly considered as key taxa in the network (Banerjee et al., 2018; Herren and McMahon, 2018).

### Lung Microbiota Varied With the Characteristics of TB Patients

**Figure 4:**
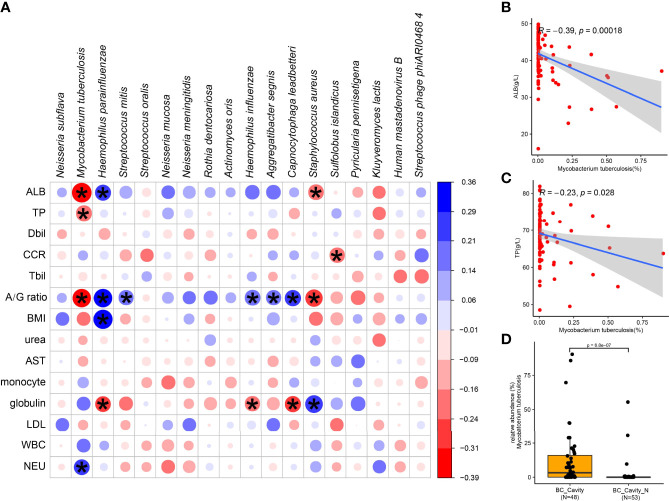
Specific microbial signatures are associated with the clinical data. The heat map shows the Spearman correlation between microbial species with clinical data **(A)**. Red values indicate species were negatively correlated with clinical data, while blue ones indicate the species were positively correlated with clinical data. Significant associations (adjusted *p* < 05) are indicated by asterisk. Spearman’s test showed that *M. tuberculosis* was negatively correlated with albumin **(B)** and total protein **(C)**. In the BC patients, the relative abundance of *M. tuberculosis in* those with cavity was significantly higher than in those without cavity **(D)**. ALB, albumin; TP, total protein; Dbil, direct bilirubin; Tbil, total bilirubin; A/G ratio, albumin/globulin ratio; BMI, body mass index; AST, aspartate transferase; LDL, low density lipoprotein; WBC, white blood cell; NEU, neutrophil; CCR, creatinine.

To further investigate the correlation between clinical characteristics and the lung microbiota of TB, we performed Spearman’s rank-based correlation test on the 18 species of microbes whose relative abundance differed significantly between the two groups (**Figure 4A**). Albumin, globulin, and albumin/globulin ratio were the main clinical factors that were significantly associated with specific species of bacteria. It is worth noting that, *M. tuberculosis* was negatively correlated with albumin (**Figure 4B**), total protein (**Figure 4C**) as well as the albumin/globulin ratio and positively correlated with neutrophil (NEU). Another species enriched in the BC group, *S. aureus*, was also negatively correlated with ALB and the albumin/globulin ratio. Accordingly, the species enriched in the BC group, such as *H. parainfluenzae*, was positively correlated with albumin and the albumin/globulin ratio. These observations suggest the dynamics of lung microbiota caused by *M. tuberculosis* is associated with the physical conditions of the patients.

*M. tuberculosis* was positively related to cavity in BC group (*p*-adjust < 0.05). In the BC group, the relative abundance of *M. tuberculosis* was significantly higher in the patients with cavity than those without cavity (**Figure 4D**), confirming again the role of *M. tuberculosis* in the formation of chest cavity.

**Figure 5:**
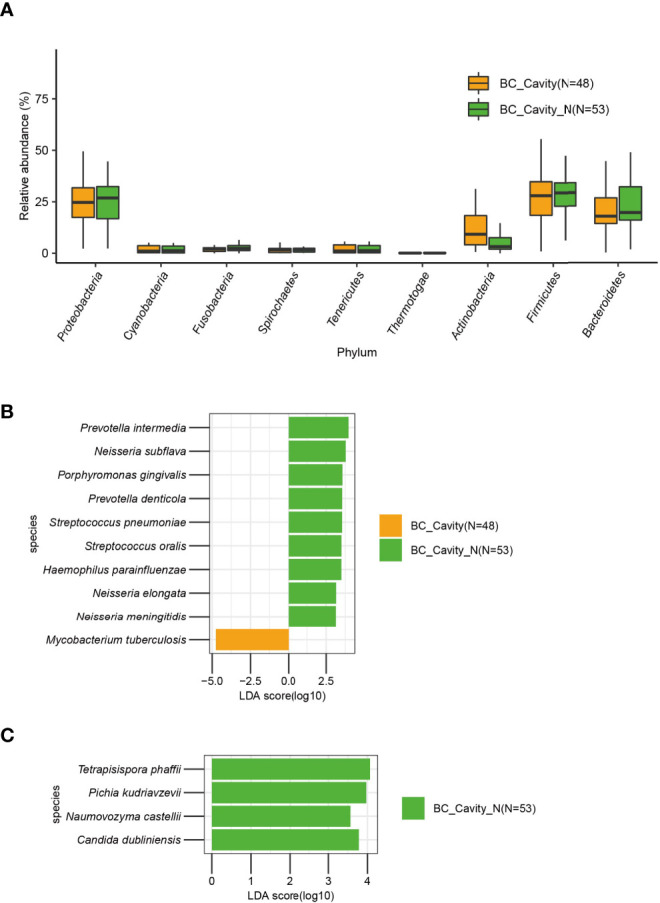
In BC patients, the presence or absence of chest cavity was significantly associated with the lung microbiome. In the BC group, *Actinobacteria* was significantly enriched in patients with chest cavity **(A)**. Bacteria **(B)** that were significantly differentially enriched in BC patients with or without chest cavity were identified by LEfSe. Fungi that were significantly differentially enriched in the BC_Cavity_N group were identified by LEfSe **(C)**.

Next, we categorized the BC patients as BC_Cavity (N = 48) and BC_Cavity_N (N = 53) as explained above. The phylum *Actinobacteria* was significantly enriched in BC_Cavity patients (**Figure 5A**). At the species level, *M. tuberculosis* was enriched in the BC_Cavity group, while *Prevotella intermedia*, *Neisseria subflava, Porphyromonas gingivalis*, and other seven bacterial species were significantly enriched in the BC_Cavity_N group (**Figure 5B**). Of fungi, *Tetrapisispora phaffii*, *Pichia kudriavzevii*, *Naumovozyma castellii*, and *Candida dubliniensis* were enriched in the BC_Cavity_N group (**Figure 5C**).

## Discussion

In this study, we improved the bacteriological confirmation approach by integrating the traditional culture methods and mNGS, leading to a higher rate of bacteriological confirmation (101 of 123 in this cohort, 82%). Compared with the culture-based methods (Chiu and Miller, 2019), mNGS has the capacity to detect all pathogens in clinical samples theoretically (Han et al., 2019) and thus, is more sensitive and more suitable for detecting difficult-to-culture microbes, including Mtb (Miao et al., 2018; Sulaiman et al., 2018). To date, several studies have provided a glimpse into the potential of mNGS in clinical and public health settings. The first case of clinical application of mNGS was the diagnosis of neuroleptospirosis in a 14­year­old severely ill boy (Wilson et al., 2014); in this case, mNGS provided the actionable information to guide effective targeted antibiotic treatment and finally led to the recovery of the patient. Since then, mNGS has been playing an increasingly important role in the diagnosis of unknown-pathogen diseases (Simner et al., 2018; Chiu and Miller, 2019). Here we improved the bacteriological confirmation rate to 82%; however, the remaining 18% of patients who had been clinically diagnosed with TB still cannot be bacteriologically confirmed. We therefore further analyzed the metagenomics data to explore the microbial features that are associated with disease phenotypes.

BALF was collected directly from the LRT and lungs and avoided contamination from the oral microbiota (Man et al., 2017; Naidoo et al., 2019). Till now, a few studies have used BALF to study lung bacteria associated with TB using 16S rRNA sequencing ( Zhou et al., 2015; Segal et al., 2017; Hu et al., 2020). In this study, we profiled the comprehensive lung microbiota of the TB patients using mNGS and found *Bacteroidetes*, *Firmicutes*, and *Proteobacteria* were the three dominant phyla in TB lungs. Of them, *Proteobacteria* was significantly enriched in the BN group; specifically, of this phylum, *H. parainfluenza*, *Neisseria subflava, and Aggregatibacter segnis* were enriched in the BN group. In contrast, *M. tuberculosis* and *S. aureus* were enriched in BC group. *S. aureus* is a major opportunistic pathogen that reside mainly in the respiratory tract as well as on the skin (Tong et al., 2015) and can cause a wide variety of clinical manifestations, such as *Methicillin-resistant S. aureus* (MRSA) infection (Hassoun et al., 2017) and inflammatory response in the blood (Labandeira-Rey et al., 2007). Serious clinical outcomes of *S. aureus* co-infection with Mtb had been reported (Manigandan et al., 2013; Lamas et al., 2019). Lamas et al. reported that *S. aureus* was co-infected with Mtb in a clinical case of primary purulent pericarditis (Lamas et al., 2019). Cadena et al. found that the *Staphylococcus* genus was enriched in the Mtb-infected macaque based on 16S rRNA sequence analysis (Cadena et al., 2018). In our study, *S. aureus* was detected in 86% of BC patients and 73% of BN patients. However, no clinical symptoms reported in TB patients were statistically significantly associated with the presence of *S aureus*. To explore the reasons for this discrepancy, we further tested the correlation between *S. aureus* and other microorganisms, and we found that *S. aureus* was positively correlated with high incidence of *Staphylococcus phage*. Usage of *S. phage* had been reported to treat the antibiotic-resistant *S. aureus* (Azam and Tanji, 2019), and thus *S. phage* may have the potential of limiting the overgrowth of *S. aureus*. This well-tuned balance between the phage and host bacteria may be the reason why *S. aureus* has a high incidence in TB patients but not many people show symptoms.

We also noticed the gender ratio issue of TB patients. In our data, the sex ratio (male/female) in the BC group was 2.48 (72/29), while in the BN group the ratio was 1.75 (14/8). These numbers are consistent with the WHO TB report (2020), in which the sex ratio for all ages ranged from 1.3 in the Eastern Mediterranean Region to 2.1 in the European and Western Pacific regions. We also compared whether sex differences would be associated with microbiome characteristics and found that *Aggregatibacter segnis* and *Fusobacterium nucleatum* showed enrichment in females in the BN group among strains with a mean relative abundance greater than 1%, while, compared to the BC group, *A. segnis* was relatively enriched in the BN group compared to the BC group.

Fungi were an unneglectable part of the lung microbiome. Due to lack of clear clinical symptoms, it was difficult to diagnose lung fungal infection, leading to high morbidity and mortality (Amiri et al., 2018); for example, *Candida albicans* may cause infections in immunosuppressive patients, and a culture-based study showed that 4.62% of TB patients were co-infected with *C. albicans* (17). In contrast, using mNGS we found that 82 BC patients (81.19%) and 15 BN patients (68.18%) in our cohort were co-infected with *C. albicans*. Our results demonstrated the sensitivity of mNGS is significantly higher than that of culture in detecting fungus. Archaea mainly live in the extreme conditions, such as high heat and pressure environment, alkaline lakes (Takai et al., 2008). Recent studies suggested that archaea also inhabits the human body, such as nose, gut, and skin (Pennisi, 2017; Sereme et al., 2019). Sereme et al. summarized in a review article that methanogenic archaea can trigger the activation of immunity system to generate specific T and B cells in human, modulating the release of antimicrobial peptides (Sereme et al., 2019). The identification of archaea in the lung of TB patients advances our understanding of the biological adaptations of archaea, although the effect of archaea in the lung of patients with TB is unknown. Mtb with other microbes together constitutes the lung microbial ecosystem of the TB patients, shaping the host immune system (Trauer et al., 2018). We found that microbial interactions were significantly reduced in the BC group, indicating that TB infection disturbed the microbial ecosystems of the lungs. The relative abundance of Mtb was associated with the pulmonary cavity in TB patients, which was consistent with previous reports (Hunter et al., 2014). One of our very important results was the finding that the abundance of *Mtb* was negatively correlated with albumin (ALB) (**Figure 4B**). Tuberculosis is a chronic infection disease, and long-term chronic infection of *M. tuberculosis* will lead to chronic metabolic deficit and increased depletion of ALB. Serum ALB level is an important marker of the nutritional state of a patient. Low ALB level often indicates the presence of malnutrition, which is closely related to the incidence of tuberculosis (Ramakrishnan et al., 2008) and low ALB levels have been shown to be a predictive risk factor for in-hospital mortality from tuberculosis (Okamura et al., 2013). On the other hand, it is also possible that the negative correlation between *M. tuberculosis* and ALB is due to the infection of *M. tuberculosis-*induced inflammatory cytokines that increase the synthesis of acute phase reactants in the liver thereby decreasing the synthesis of ALB; this hypothesis warrants further studies to test it.

Although we performed a comprehensive analysis of the TB lung microbiota, there were certain limitations in this study. Since it was a retrospective study, all microorganisms were profiled by sequencing the frozen DNAs that were previously extracted and stored. Thus, some specific microorganisms, such as RNA virus, may not be detected by this mNGS approach.

## Conclusions

In summary, our study demonstrated the great potential of mNGS for early and accurate diagnosis of TB patients, which leads to an effective targeted therapy for improved clinical outcomes. The comprehensive profiling and comparison of the lung microbiota between the BC and BN patients provided a new perspective for further understanding the various phenotypes and pathogenesis of tuberculosis.

## Data Availability Statement

The original contributions presented in the study are publicly available in the China National Center for Bioinformation: https://bigd.big.ac.cn/gsa/browse/CRA004908.

## Ethics Statement

The studies involving human participants were reviewed and approved by The Institutional Review Board of the Fifth Affiliated Hospital, Sun Yat-sen University. Written informed consent to participate in this study was provided by the participants’ legal guardian/next of kin.

## Author Contributions

Study concept and design: TD, LD, XL, and JX. Acquisition of data: LD, XL, XQL, and HO. Analysis and interpretation of data: YL, TD, and XW. Drafting of the manuscript: YL and TD. Critical revision of the manuscript for important intellectual content: TD, YL, and MW. Statistical analysis: YL and LD. Administrative, technical, or material support: TD, JX, and XL. All authors contributed to the article and approved the submitted version.

## Funding

This work was supported by Guangdong Key Field Research and Development Plan (2019B020228001), Guangdong Basic and Applied Basic Research Foundation [2020A1515010255], Fundamental Research Funds for the Central Universities (19ykpy43), Open project of Key Laboratory of Tropical Disease Control (Sun Yat-sen University), Ministry of Education (2020kfkt08), the Medical Science and Technology Research Fund of Guangdong (A2018275). Additional grants were obtained from Zhongshan School of Medicine, Sun Yat-sen University.

## Author Disclaimer

The DNA extraction, DNA library construction, and sequencing were performed by BGI, China. BGI had no influence on study design, data collection, analysis, decision to publish or preparation of the manuscript.

## Conflict of Interest

The authors declare that the research was conducted in the absence of any commercial or financial relationships that could be construed as a potential conflict of interest.

## Publisher’s Note

All claims expressed in this article are solely those of the authors and do not necessarily represent those of their affiliated organizations, or those of the publisher, the editors and the reviewers. Any product that may be evaluated in this article, or claim that may be made by its manufacturer, is not guaranteed or endorsed by the publisher.
