# Supplementary material for: Pathogen Metagenomics Reveals Distinct Lung Microbiota Signatures Between Bacteriologically Confirmed and Negative Tuberculosis Patients
Source: Front Cell Infect Microbiol. 2021 Sep 13;11:708827. doi: 10.3389/fcimb.2021.708827 (PMC8475726; doi:10.3389/fcimb.2021.708827)
Supplement: Supplementary file 1 [file DataSheet_1.docx]

**Figure S1. The lung microbial signature differs between BC and BN patients.**

*Euryarchaeota*, *Crenarchaeota*, and *Thaumarchaeota* were the three major phyla of archaea among TB patients(A). The relative abundance of Sulfolobus islandicus, which belonged to Crenarchaeota, was differed significantly enriched in BC group between the two groups (B). *Siphoviridae* was the most abundant family in the lung of patients with TB. (C).
