# Supplementary figures and images for: Pathogen Metagenomics Reveals Distinct Lung Microbiota Signatures Between Bacteriologically Confirmed and Negative Tuberculosis Patients
Source: Front Cell Infect Microbiol. 2021 Sep 13;11:708827. doi: 10.3389/fcimb.2021.708827 (PMC8475726; doi:10.3389/fcimb.2021.708827)

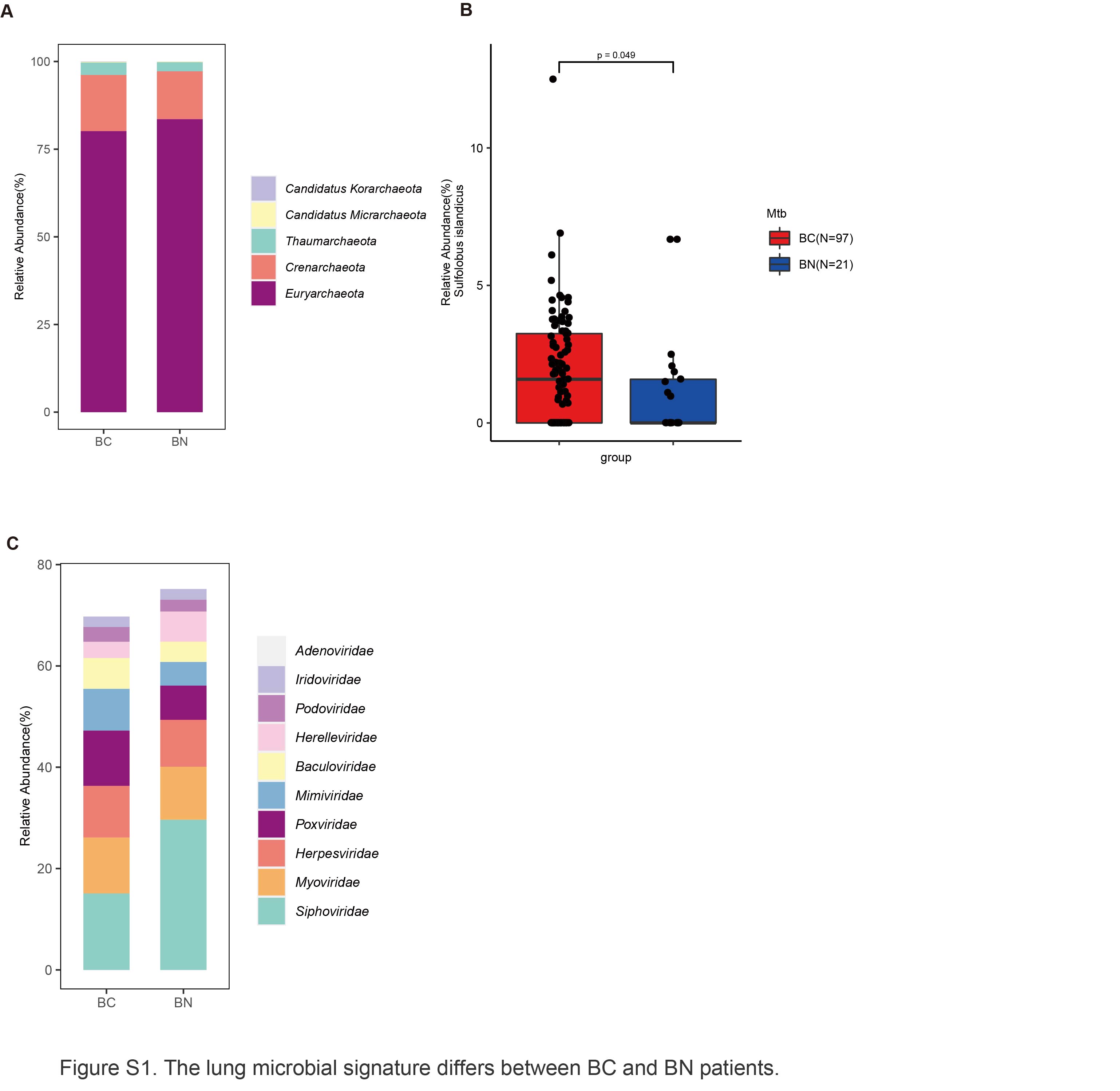

Supplement: Supplementary file 2 [file Image_1.jpeg]
